# Disability and the household context: Findings for the United States from the public Use Microdata Sample of the American Community Survey

**DOI:** 10.3389/fresc.2022.875966

**Published:** 2022-08-12

**Authors:** Christiane von Reichert

**Affiliations:** ^1^Department of Geography, Franke College of Forestry and Conservation, University of Montana, Missoula, MT, United States; ^2^RTC: Rural at the Rural Institute for Inclusive Communities, University of Montana, Missoula, MT, United States

**Keywords:** disability, persons without disability in households with disability, single-person households, family solidarity, capability approach, urban-rural, American Community Survey, Public Use Microdata Sample (PUMS)

## Abstract

**Introduction:**

Based on questions about impairments and activity limitations, the American Community Survey shows that roughly 13% of the U.S. population is experiencing disability. As most people live in households with other persons, this study explores disability at the household level. Considering the literature on household decision-making, solidarity, and capabilities in disability, this analysis of the household context of disability takes into account residential settings, household composition, and urban–rural differences.

**Method:**

The 2015–2019 ACS Public Use Microdata Sample (PUMS), which shows persons with disability (PwD) and persons without disability (PwoD), also indicates household membership, used here to separately identify PwoD as those living in households with persons with disability (PwoD_HHwD) and those in households without any household member with disability (PwoD_HHwoD). Relationship variables reveal the composition of households with and without disabilities. An adaption of Beale's rural–urban continuum code for counties is used to approximate rural–urban differences with ACS PUMS data.

**Results:**

Solo living is two times as common among persons with disability than among persons without disability, and higher in rural than urban areas. In addition to 43 million PwD, there are another 42 million PwoD_HHwD. Two times as many persons are impacted by disability, either of their own or that of a household member, than shown by an analysis of individual-level disability. For family households, differences in the composition of households with and without disabilities are considerable with much greater complexities in the makeup of families with disability. The presence of multiple generations stands out. Adult sons or daughters without disability play an important role. Modest urban–rural differences exist in the composition of family households with disability, with a greater presence of multigenerational households in large cities.

**Discussion:**

This research reveals the much wider scope of household-level disability than indicated by disability of individuals alone. The greater complexity and multigenerational makeup of households with disability imply intergenerational solidarity, reciprocity, and resource sharing. Household members without disability may add to the capabilities of persons with disabilities. For the sizeable share of PwD living solo, there is concern about their needs being met.

## Introduction

Data from the American Community Survey (ACS) show that, based on physical impairments and functional limitations, nearly 43 million or 13% of the U.S. population experience disability. Disability rates vary by location and are generally lower in large metropolitan counties and a good deal higher in very rural counties and in the southern parts of the United States (1).

The ACS captures disability as a binary attribute of persons: persons with disability (PwD) and persons without disability (PwoD). Of persons with disability, 20% live solo and another 7% live in group quarters. However, most persons with disability live in households with other persons, predominantly in family households (2). In these residential settings, other household members may also experience disability, but more commonly, they are persons without disability. Without disability of their own, these persons nonetheless are impacted by disability through that of another household member. Quite likely, the lives of persons without disability in households with disability are different than the lives of persons without disability in households without disability, simply because of the nature and makeup of the household.

Microdata from the ACS show attributes of persons and household membership, and this allows for an analysis of disability of persons in their household context. Specifically, PwoDs are separated into two groups: those living in households with another household member experiencing disability (PwoD_HHwD) and those without other household members with disability (PwoD_HwoD). Using this distinction, and as laid out below, this analysis of ACS microdata reveals that, besides 43 million persons with disability of their own, there are over 42 million persons without disability living in households with disability.

There is good rationale for considering the household setting when examining disability, as household members live and act in the context of their household. Three conceptual approaches provide a background for this analysis: household decision-making, household solidarity, and capabilities in disability.

Decision-making is shaped by household membership, as highlighted by Becker (3, 4) and Mincer (5, 6). Households, especially family households, function as units with decisions made at the household level. Household decisions are centered on what benefits the household, even at the expense of an individual household member. Household decisions involve the use of time, labor force participation, migration, health, and more. For households with disability, the concept of household decision-making suggests that the needs of a household member with a disability could impact available options and choices for persons without disabilities in these households. Persons without disabilities in households with a disability may be involved in caregiving and could very well face different opportunities and make different choices than their counterparts in households without disabilities. Indeed, U.S. persons without disabilities in households with disability showed lower migration rates during young adulthood. As young adulthood is a period of peak migration, reduced migration rates during these peak years can translate into lower lifetime mobilities (7). In a literature review of young caregivers in Australia, Day (8) expressed concern about significantly reduced future life opportunities for this cohort, while acknowledging both the challenges and rewards that come with caregiving.

Solidarity in households, in particular family households, indicates that households are units of support. Households may exercise same-generation and intergenerational solidarity (9–12). Same-generation support between spouses and partners tends to be the norm. Siblings are also of the same generation. Intergenerational support (between generations) tends to go from parents to children (forward support to the next generation) or adult sons/daughters to elderly parents/parents-in-law (backward support to the previous generation). In a household with disability, where support relationships are crucial, it has been shown children and young adults provide care for parents or grandparents, thereby reversing roles and the standard intergenerational directions of support. A 2019 survey conducted by the National Alliance of Caregiving and the American Association of Retired Persons reveals a growing number of teenage and young adult grandchildren (who could be resident members of the household or non-resident) are caregivers for grandparents (13).

As part of these solidarities, households/families also function as economic units: Household members living under the same roof can share their resources, such as income or housing assets, or split household expenses. Pooling of household resources may be especially relevant for persons with disability. Persons with disability face diminished opportunities to participate in the labor force (14). In addition to these indirect costs of disability, there are sizeable extra direct costs of living with a disability (15). Higher indirect and direct costs of disability, along with lower incomes (16), translate into a greater likelihood of being in poverty (17). In essence, persons with disability may have greater resource needs while faced with limited household resources to meet those needs. Additional members of the household could be positioned to fill that gap between needs and resources. Sen (18) referred to household resources as “commodities” and proposed a capability approach to human wellbeing, later adapted by Nussbaum (19), Mitra (15), Trani, and Dubois with co-authors (20, 21) and others to better and differently conceptualize disability.

“Capabilities” or practical opportunities make for a set of choices that affect people's “functioning” or actually achieving what they value. Besides “commodities,” capabilities are shaped by the environments people live in and their personal characteristics, such as age, education, and impairments (15, 20). Disability results from deprivation in capability and/or functioning. Impairments are “potentially disabling” but only “actually disabling” if they restrict people's capabilities or functioning, meaning they bar people from doing what they would like to do or value [(15), p. 241]. In household settings, capabilities of individuals may be increased with others contributing or sharing their capabilities, such as caregivers (22). Beyond the household, members of the community people live in may also offer support. Consequently, individual capabilities can be enhanced and turn into collective capabilities [(20), p. 8, (23)].

The merit of the capability approach for this analysis lies in looking at the household as a unit for increasing capabilities and reducing potential disability. In other words, the presence of household members without disability quite likely improves the lives of persons with impairments or activity limitations. PwoD_HHwD provide PwD more opportunities to fully participate in their community. At the same time, the presence of household members with disability also affects in major ways the capabilities and functioning of persons without disability sharing the household.

ACS microdata on disability used in this research do not offer explicit insight on household decision-making, household support, and impacts on lives based on capabilities and functioning. However, the large sample of the ACS Public Use Microdata Sample PUMS can be used to identify the residential setting (group quarter, solo, family households, and non-family households with two or more people) of PwD and PwoD and to describe in depth the composition of households with or without members with disability. The relationships between household members (reference person, spouse/partner, son/daughter, elderly parent/parents-in-laws, and grandchildren) with or without disability indicate caregiving relationships and resource sharing with the potential to affect capabilities and functioning. Capabilities made available through household members can expand opportunities for persons with impairments and functional limitations, thereby increasing activity and favorably affecting community participation.

Using a three-way classification of disability (PwD, PwoD_HHwD, and PwoD_HHwoD), this research sets out to identify the residential setting for persons with and without disability, describe the composition of households with and without disability, with special emphasis on family households, and present data for the nation and separately for areas along the urban–rural continuum.

The merit of this analysis lies in expanding the analysis of disability by considering the household context, to better identify the scope of disability and the residential setting in which it occurs, with a focus on the composition of family households where over 80% of the U.S. population live.

Beyond analyzing national-level data, an analysis of urban–rural differences in residential settings and household composition is called for because of sizeable differences in the rural–urban disability rates. Higher rates of disability in rural than urban areas were found to be accompanied by a shortage of formal support and caregivers in rural areas (24). This service gap may lead to a greater need for household members (as well as non-resident family and friends) to provide support and caregiving to persons with disability. Supportive rural environments may reduce restrictions in the lives of people with impairments and activity limitations by supplementing individual capabilities with collective capabilities. Alternatively, if collective capabilities are limited, rural people with impairments may lead more restricted lives, especially if living solo.

## Methods

This descriptive analysis is based on the Public Use Microdata Sample (PUMS) of the 2015–2019 American Community Survey (ACS) (2). Five-year data are used to benefit from the larger sample size, which consists of over 6.2 million households (HH) with nearly 15.2 million people plus 750,000 persons in group quarters (GQ). By applying person weights to extrapolate from persons in the sample to the U.S. population, the 2015–2019 ACS PUMS puts the U.S. population at 325 million.

Based on the ACS definition of disability, there are nearly 43 million persons with disability in the United States and a disability rate of 13%. In the ACS, disability is a binary variable (present or absent, 1 or 0) based on self-reported binary responses to four impairment questions (ambulatory or walking, cognitive, vision, and hearing) and two functional limitation questions (self-care and independent living). Disability is deemed to be present if a person answers affirmative to at least one of the six questions. Multiple affirmative responses are quite common (two or more impairments or limitations account for 75% of persons with disability), with ambulatory impairment and independent living limitation being the most common combination followed by independent living limitations and cognitive impairment. There is some overlap in the ACS questions with the six questions from the Washington Group on Disability Statistics Short Set (WGSS) (seeing, hearing, walking, cognitive-remembering, self-care, and communication) (25). However, in contrast to the binary measure of the ACS, the WGSS uses a Likert scale to develop a disability score, therefore an ordinal measure of disability. The WGSS, developed for international use in general population surveys or censuses, where a limited number of questions can be asked on a wide range of attributes, was designed to capture the majority of people with activity limitations that most often restrict participation (26). The WGSS is informed by the World Health Organization's 2001 International Classification of Functioning, Disability, and Health ICF [(27), p. 5]. The ICF presents disability as impairments giving rise to activity limitations and participation restrictions [(15), p. 238].

Disability definitions have changed and are evolving (15, 20, 28, 29), demonstrating that disability is multifaceted and complex. While ACS questions and definitions on disability cannot fully capture that complexity, the ACS questions have merit as shown by their overlap with the Behavioral Risk Factor Surveillance System (BRFSS) (30), the nation's premier health-related survey. Importantly, the ACS is, by design, a very large sample of the U.S. population with an extensive set of variables. Variables include household membership and household relations used here to analyze residential settings and household composition. In contrast to smaller surveys which work well at the national level, the sample size of the ACS further allows a breakdown of rural–urban differences in disability and household composition. Disability as a three-way (PwD, PwoD_HHwD, and PwoD_HHwoD), not binary (PwD and PwoD), classification of persons in households relies on three variables of the ACS: the serial number of the household a person belongs to (SERIALNO), the relationship variable which shows the relationship of household members to the reference person (person who answered the survey, presumably the “householder,” RELSHIPP), and the disability variable (DIS) (31). The reference persons may also live solo (single-person household). Populations in institutionalized and non-institutionalized group quarters are identified as well, but not considered to be members of a household.

These variables make it possible to separately identify persons without disability (PwoD) as those living in households of two and more with (1) another person/s with disability (PwoD_HHwD) or (2) other household members without disability (PwoD_HHwoD). Household affiliation, relationship, and disability variables are used to identify the residential setting, and the composition of households with and without disability. Table S1 in the Supplement illustrates the process of transitioning from the binary disability variable of the ACS to a three-way classification of disability at the household level used here. The three-way classification allows pinpointing who persons with disability live with and how the household compositions differ for households with or without disabilities. Additional variables, such as marital status, subfamilies, or race, were consulted as well for select data queries.

To explore urban–rural differences in household compositions, a measure of urbanity–rurality is needed. ACS PUMS data are released for Public Use Microdata Areas (PUMAs), sizeable areas with a population of at least 100,000. In large metropolitan (metro) areas, PUMAs consist of census tracts, while in smaller metro and nonmetropolitan (nonmetro) areas, PUMAs contain one or several counties. However, PUMAs are not published showing a measure of urbanity or rurality. The Census Bureau also designed MIGPUMAs, to track migration, (as well as POWPUMAs, to track places of work) using counties as building blocks (32). For counties, two widely used urban–rural classifications exist: the metro-micro-noncore classification of the Office of Management and Budget (33) and the rural–urban continuum or Beale code of the Economic Research Service (34). The urban–rural measure employed here builds on the Beale code of counties that make up MIGPUMAs weighted by county populations. The urban–rural code assigned to MIGPUMAs consists of eight categories, ranging from most highly urbanized (large metro) to most rural (nonmetro-highly rural). While an approximation, the urban–rural continuum code of MIGPUMAs derived from county Beale codes does well in replicating the urban–rural distribution of the resident population and disability rates of counties shown in ACS 2015–2019 summary data (35).

## Results

### Overview: Residential settings of persons with or without disability

Nearly 43 million (13%) Americans with disability live in various types of residential settings: in non-institutionalized and institutionalized group quarters, in single-person (solo) households, or in households of two or more persons as family or non-family households.

Population shares and disability rates vary by residential setting. Persons in group quarters account for <3% of the U.S. population but close to 7% of persons with disability due to disability rates of 18% in non-institutional group quarters and 54% in institutional group quarters. Persons in single-person households represent 10% of the U.S. population, but 20% of persons with disability, making solo living twice as common among persons with disability, thereby upping the disability rate of solo households to 26%, twice the U.S. average. Family households account for 81% of the U.S. population, and non-family households account for 6%, with disability rates around 11% (Please see Table 1).

**Table 1:** Residential settings of the U.S. population, persons with disability (PwD), and persons without disability in households with disability (PwoD_HHwD).

	**U.S. population**	**Persons with disability**		**Persons without disability in households with disability**	
Residential settings	Persons^a^ (%)^b^	Persons^a^ (%)^b^	Rate^c^	Persons^a^ (%)^2^	Rate^d^
Non-institution. GQ^e^	4.2 (1.3)	0.7 (1.7)	17.7		
Institutional GQ^f^	3.9 (1.2)	2.1 (4.9)	53.6		
Single-person HH^g^	33.6 (10.4)	8.7 (20.4)	25.8		
Family HH^g^	262.8 (80.9)	28.8 (67.6)	10.9	40.3 (95.4)	15.3
Non-family HH^g^, ^h^	20.2 (6.2)	2.3 (5.5)	11.5	1.9 (4.6)	9.6
All settings in the U.S.	324.7 (100.0)	42.6 (100.0)	13.1	42.2 (100.0)	13.0

a Persons in million.

b Percent of U.S. population.

c Rate of PwD (disability rate) for residential setting.

d Rate of PwoD_HHwD for residential setting.

e Group quarters including dorms, military barracks, and group homes.

f Group quarters including nursing homes, correctional facilities, and mental hospitals.

g HH households.

h with two or more persons.

Source: Christiane von Reichert, derived from the 2015-2019 American Community Survey Public Use Microdata Sample (ACS PUMS).

Table 1 also shows summary data for PwD and PwoD_HHwD by residential settings (PwoD_HHwoD account for the remainder and are not shown to avoid redundancy). There are 42 million PwoD_HHwD, which per definition only include households with two or more persons. Their number is just slightly less than the number of PwD in the United States. In family households, however, the number of PwoD_HHwD is at 40 million larger than the number of PwD at 29 million.

By recognizing persons without disability in households with disability, disability—either their own disability or that of a household member—affects more persons than binary measures of disability capture. ACS PUMS data show there are twice as many persons impacted by disability at the household level than disability rates for individuals suggest. The three-way classification of disability shows up as especially important in family households where disability experiences are widely felt and broadly shared.

The household literature suggests that individual members of a household may be tied in their decisions to household needs and act out of solidarity with other household and family members. In households, especially in family households with disability, the needs of PwD may influence everyday living and major household decisions. In this context, PwoD_HHwD feel the impact of disability through the presence of another household member with disability, and their life is shaped by disability at the household level. This analysis shows that the number of PwoD_HHwD is sizeable.

### Residential settings of persons with or without disability along the urban–rural continuum

**Figure 1:**
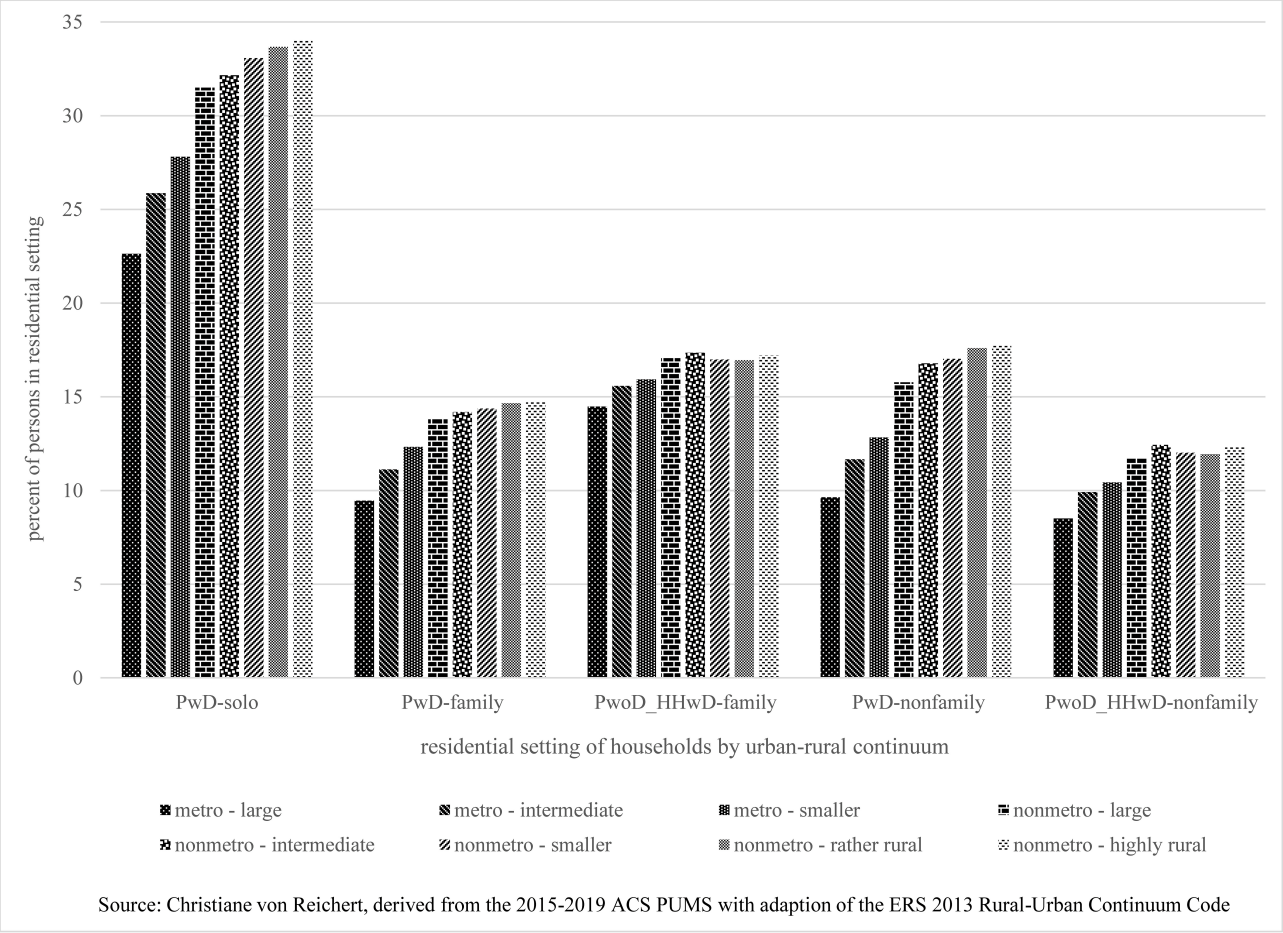
Persons with or without disability in single-person, family, and non-family households by urban–rural continuum.

Urban–rural differences in rates of disability are well recognized with higher rates of rural than urban disability. The 2015–2019 ACS PUMS data put disability rates in large metropolitan areas at 11% with rates stepping up for smaller metro and larger nonmetro areas and rising further to 18% for highly rural areas, much above the national average of 13% (see Figure 1 below).

While this geographic pattern is not that evident for the relatively small group quarters population (not shown in Figure 1), urban–rural differences are very much apparent for solo households, where disability rates of single-person households are 23% in the most highly urbanized areas and 34% in the most rural areas. These high disability rates of solo households are striking but especially concerning for highly rural areas, where health and social services tend to be limited and formal caregiving is disturbingly low (24). Higher rural disability rates could stem from rural areas being nonetheless more amenable to solo living of PwD as they may receive support from non-resident families, friends, and neighbors. There also could be the benefit of lower rural housing costs keeping housing affordable and within reach of a person with disability, even if single. In other words, individual capabilities could be increased through “collective capabilities” [(20), p. 398, (23)] based on support-based relationship or through a more favorable bundle of resources or “commodities” (15, 18). Both would increase capabilities, improve functioning and create incentives for living in supportive, and lower-cost rural communities. Alternatively, solo persons with disability in rural areas may not have these benefits while seeing limited other options such as living in group quarters or with family. Group quarters may be less available or hardly affordable, or close family members may have moved away, and solo persons' opportunities to move nearer to them may be constrained.

The rise of disability rates with increasing levels of rurality also holds for family and non-family households (Figure 1). Also noteworthy is the somewhat higher rural share of PwoD_HHwD in family households. Their greater share may be a benefit to these households as persons without disability may be tasked to provide informal support for family members with disability if service infrastructure and formal support are limited in more rural places. A rural focus on familism, age structure, and composition of household members could also come into play.

For non-family households, there is an even bigger increase in disability rates between urban and rural households. However, the number of non-family households is relatively small overall and particularly small in rural areas.

As it stands, while data from the ACS cannot explain the phenomenon, they clearly state that PwD and PwoD_HHwD make for a larger share of rural than urban populations. Conversely, PwoD_HHwoDs make for a smaller share of rural than urban populations, indicating that disability is experienced less in urban than rural areas.

The three-way (vs. two-way) classification of disability is therefore particularly relevant for rural areas. It highlights the even greater extent and impacts of rural disability than the binary disability classification, as disability is more widely felt by PwoD_HHwD in rural than urban areas, especially affecting rural family households.

The following segment focuses on family households and their composition.

### Differences in the composition of family households with or without disability

Family households with disability differ in important ways from family households without disability. The composition of families with disabilities is much more complex, partly as a result of including persons with and without disabilities, but also due to the different types of relationships to the reference person, and differences in age distributions.

Family households without disabilities reflect the nuclear or core family made up of a reference person with a spouse/partner or single parent along with their sons and daughters. These three groups account for the overwhelming majority, roughly 92%, of family members. The share of extended family members (elderly parents/parents-in-laws, grandchildren, or other families) is small with only 7%, plus a very small share of unrelated household members.

Family households with disability deviate considerably from the core family model: Reference person, spouse/partner, and sons/daughters account for just over 80%, while elderly parents/parents-in-laws of the reference person show up among persons with disability. Persons without disability in these households include a good number of grandchildren. There are others, consisting mostly of relatives such as brothers/sisters of the reference person, a small number of sons-in-law/daughters-in-law, other relatives, and a few nonrelatives.

The multigenerational makeup stands out as a feature of family households with disability. Extended and multigeneration households have been seen as ways of lessening economic and personal hardship (12, 36). The disability of one or several family members could be contributing to challenges that a multigenerational household can address through reciprocity of family members giving and receiving mutual support and thereby increasing collective capabilities.

**Figure 2:**
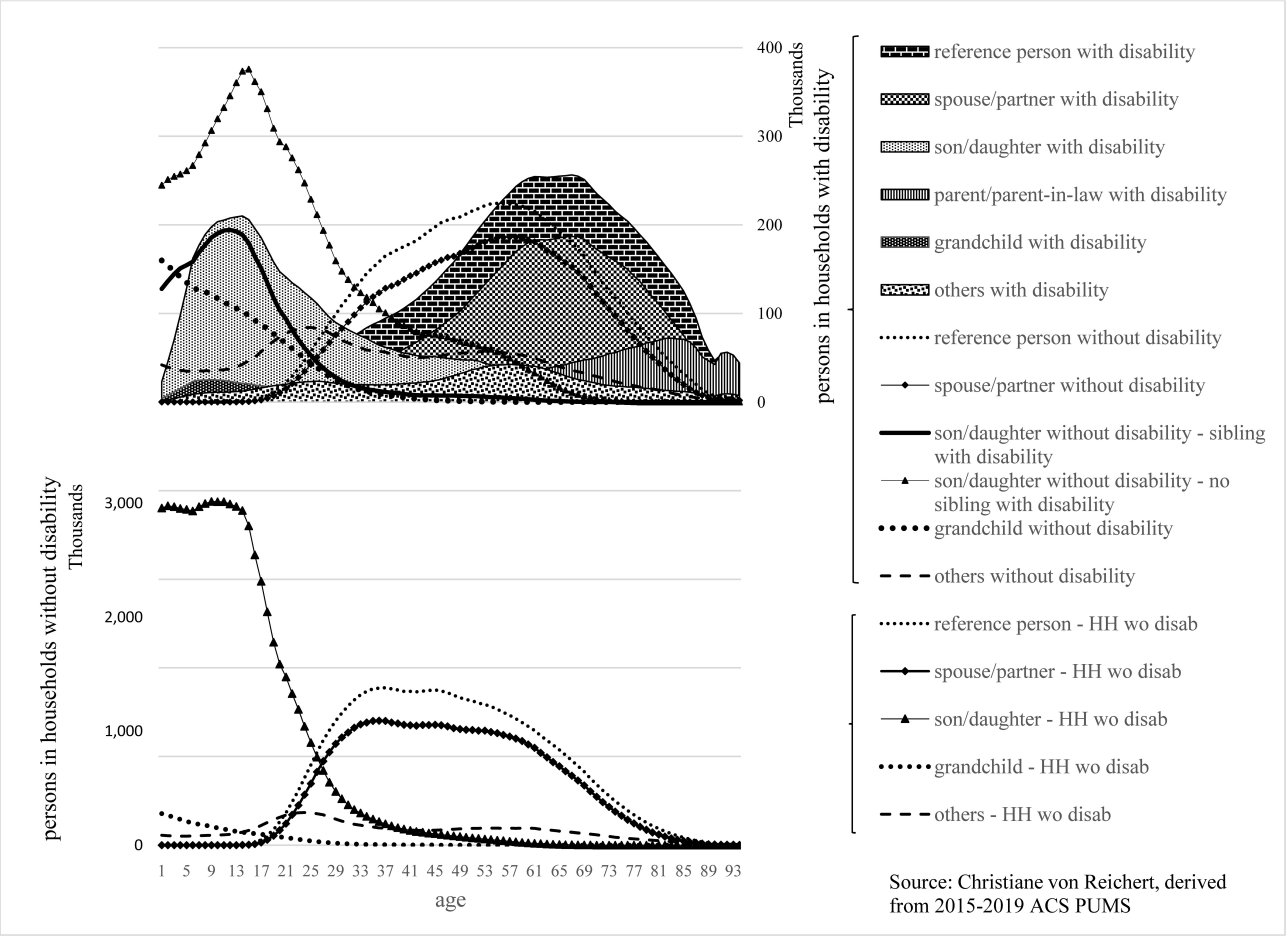
Persons in family households with and without disability: composition by relationship to reference person, disability, and age.

The composition by age and relationships of family households with and without disability is shown in Figure 2. The top segment of Figure 2 shows the composition of family households with disability, and the bottom segment represents families without disability (using five-year moving averages for a visually less distracting, smoother display). PwDs are presented as areas with patterns and PwoD as lines. The Y-axis shows the number of household members in each relationship category and disability group by single year of age (X-axis). To account for big differences in the number of persons in family household with disability and without disability, the information is displayed using two Y-axes. Differences in the complexity in the composition of households with disability *vis-à-vis* the core family composition of households without disability are pronounced.

As a note: Figure 2 shows cross-sectional ACS data and therefore a snapshot in time. Changes by age point toward life course transitions and household dynamics. Transitions taking place as people pass into higher age groups have relevance in disability analysis, as argued in the following.

In families with disability, the age of reference person and spouse/partner with disability peaks in the 60 s and for reference person and spouse/partner without disability in the 50 s. In families without disability, reference person's and spouse's/partner's age tends to peak between the mid-30 s and mid-40 s. The younger age of a reference person and spouse/partner in households without disability translates into the younger age of their sons/daughters vis-à-vis those in families with disability. For families with disability, sons/daughters of the reference person are disaggregated into two groups to account for noteworthy differences: those who have siblings with disability and those who do not. Children and adolescents without disability who grow up alongside siblings with disability may be among the most under-studied group in disability research. Yet, the impacts on their lives are profound (37), and their connection to siblings with disability may stretch over decades, longer than that of the parents (38).

In families with disability, nearly one-fourth of the reference persons' young and adolescent sons/daughters (ages 0–19) experience disability. There is a near equal number of their siblings without disability in the corresponding age groups. This means on average, children and adolescents with disability grow up with siblings without disability, and these siblings without disability encounter disability by coming of age alongside siblings with disability. A rapid drop in the number of sons/daughters without disability (with siblings with disability) in early adulthood (ages 20 to 25) suggests they leave the parental home in their early to mid-20 s, as do their counterparts in households without disability. For sons/daughters with disability, there is a decline in their number in early adulthood as well. However, that decline is much more gradual suggesting their departure from the parental home stretches into their mid-30 s. There are also sons/daughters with disability beyond age 30, indicating they live in the parental home well into adulthood, suggesting they may be staying with, not leaving, the family.

The great majority of sons and daughters without disability in family households with disability live with a reference person or the reference person's spouse/partner with disability (These are their parents/parent). In this household constellation, the number of sons/daughters without disability declines gradually in young adulthood, suggesting they leave the parental home at a relatively slow pace (or possibly returning to that home) compared to those with disabled siblings. From their mid-30 s to well into their 50 s, that group (sons and daughters without disability) account for a small but noteworthy and near constant number of household members. This suggests a certain number of adult sons/daughters without disability continue to reside into their 50, even 60 s with reference person and spouse (their parent/s with disability). A majority of those over 30 never married, and a quarter are divorced. Their choice of residence suggests support relationships between adult sons/daughters without disability and their parent/s with disability exist that may explain the much higher shares in households with than without disability. There is a sharp drop in shares around age 60. Concluding that sons/daughters without disability, who spent their adult life residing with parent/s with disability, would depart from the family home and leave aging, disabled parent/s at this stage of their life is not that plausible. More likely, the mature adult son/daughter becomes the reference person, and the previous reference person with disability shows up as a parent with disability. If this interpretation stands up, people who make up the family remain unchanged, even if relationship classifications change. The presence of the previous generation of adult sons/daughters turning into the reference person allows elderly parents with disability to continue living in the family household and age in place.

There is another angle on adult sons/daughters in their 30 s to 50 s living with an older reference person with disability. Of sons/daughters without disability over 30, over a one-fourth live in the parental home as subfamilies, some as a couple with or without children but most as a single parent with children. The children in the subfamilies of adult sons/daughters show up as grandchildren of the reference person, and two-thirds of grandchildren are children in subfamilies.

In essence, the complexity and intergenerational makeup of households with disability can be partly explained by adult sons/daughters without disability living in the family household well beyond adolescence.

Elderly parents contribute to the multigenerational mix of family households with disability. In these households, the number of elderly parents/parents-in-law with disability increases with age, as expected. This could partly be, as pointed out above, the result of previous reference persons transitioning into the parent/parent-in-law relationship class. It also could stem from the onset of disability of aging parents who have lived with the family for some time. In addition, elderly parents may move to join the family household.

Other family and a few non-family are also present in families with disability, accounting for under 8% of the members in the family household, for a one-to-two split of PwD vs. PwoD_HHwD.

### Urban–rural composition of family household with disability

**Figure 3:**
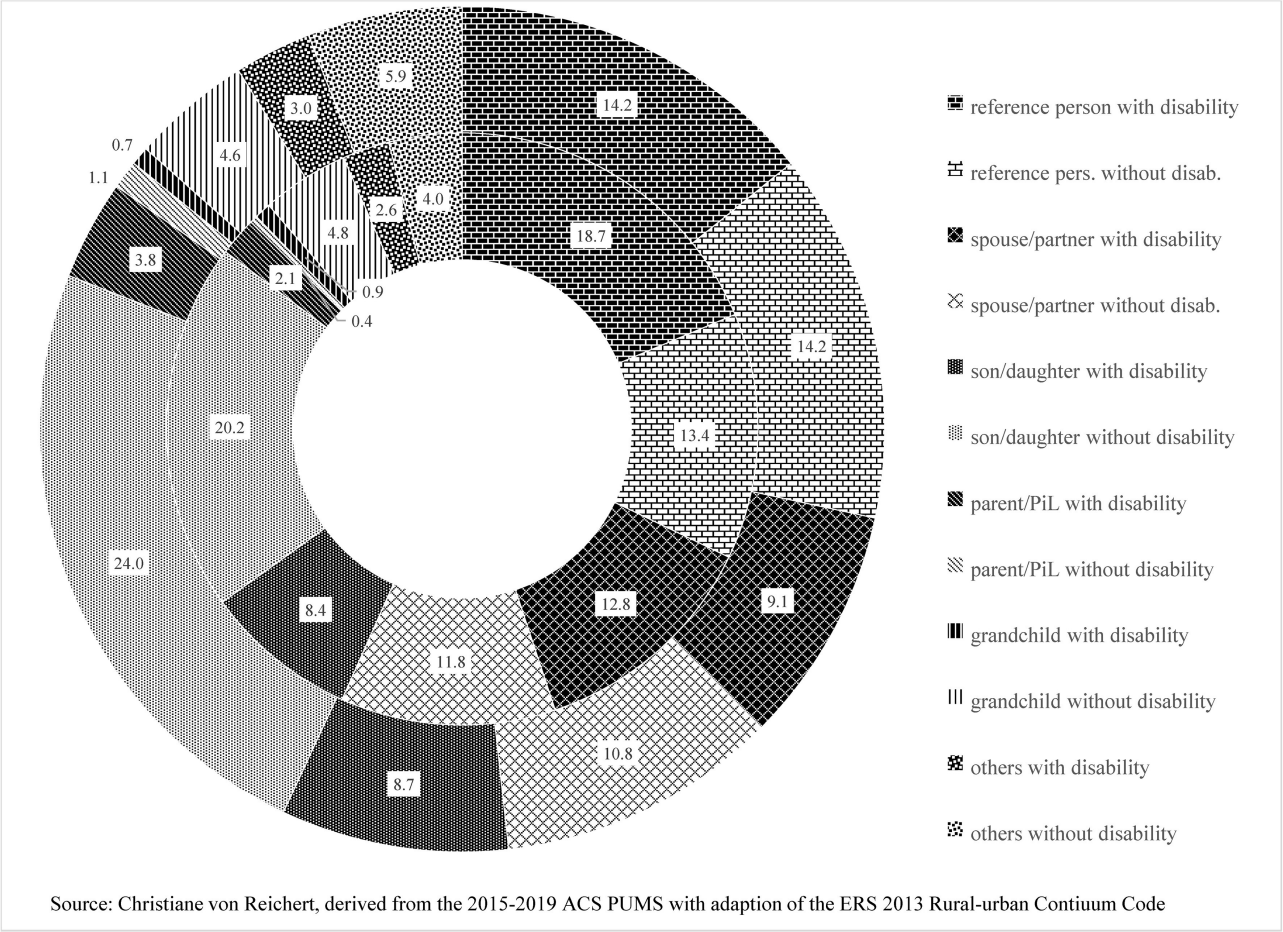
Composition of family households with disability in large metropolitan and nonmetropolitan areas.

Differences in urban and rural rates of disability and a higher share of PwoD_HHwD in rural than urban areas raise the question of whether or not urban–rural differences also show up in the composition of families with disability. It turns out that ACS microdata show relatively modest differences in the composition of family households with disability in urban (metropolitan or metro) vis-à-vis rural (nonmetropolitan or nonmetro) areas (Figure 3).

Figure 3 is a two-ring donut chart showing the composition of family households. The inner ring represents rural/nonmetropolitan areas, and the outer ring shows the most urban/large metropolitan areas. Rural/nonmetropolitan areas, from large nonmetro to highly rural, are combined due to near identical household compositions (The household composition of intermediate and smaller metro areas fall between large metro and nonmetro and are excluded in the chart for not showing much additional information).

The shares of reference persons and spouses with disability are higher in nonmetro areas than in large metro areas, and the higher nonmetro shares contribute to the higher rural disability rate. In metro and nonmetro areas alike, there are fewer spouses or partners than reference persons pointing toward families not including a spouse/partner and made up of the reference person and other related persons (son/daughter, parent/parent-in-law, and other relatives) and a very small number of nonrelatives.

The share of reference persons and spouses in metro areas is somewhat smaller than in nonmetro areas, partly a result of the greater presence of sons and daughters without disability. Factors potentially associated with their higher shares of sons/daughters are the younger age of metropolitan reference persons (with fewer empty-nesters), the greater presence of single-parent households, along with greater racial and ethnic diversity in highly urbanized areas.

Parents/parents-in-law represent a small share of household with disability. There is, however, a greater presence of parents/parents-in-law with disability in larger metro areas than in nonmetro areas. The higher shares in metro households could be the flipside of less solo living in urban than rural locales. The lower share of parents/parents-in-laws with disability in rural households may indicate that the option of living with close family is perhaps less available if adult children and their families left rural for urban areas, as part of a long-term population trend. The larger share of PwD living solo in more rural areas lends support to that argument.

For grandchildren, there are near equal shares in nonmetro and metro family households with disability. The small share of other family household members (brothers–sisters of reference person, some other relatives, and very few nonrelatives) without disability is somewhat larger in more urban than more rural areas, which also contributes to the somewhat larger metro family size.

Analyzing the composition of a family household with disability shows that nonmetro and metro areas alike have highly complex household relationships which much deviate from the core family model of families without disabilities. Across the nonmetropolitan categories, differences appear to be minimal. However, in larger cities, the complexity is greater than for nonmetro households. Additional queries for racial groups (not shown here) reveal that the greater ethnic and racial diversity in larger metropolitan areas contributes to that complexity presumably due to a larger role of extended family and multiple generations, especially for families with disability. The multigeneration household appears to be a strategy to cope with higher costs and greater resource needs associated with disabilities. Additional household members sharing incomes and assets, such as housing, add to household resources or “commodities.” The “collective capabilities” may receive a boost in these multigeneration households.

## Discussion

Using microdata from the 2015–2019 American Community Survey, this research builds on information about relationships in households and the residential setting of persons with and without disabilities. Leaving group quarters mostly aside, residential settings are separated into single-person (solo) households and two-plus person family households or non-family households.

For two-plus person households, most of which are family households, this research draws attention to a group often unrecognized in disability research and policy: persons without disability in households with disability. This common oversight stands in stark contrast to the major role many PwoD_HHwDs play in the lives of persons with disability. Based on the ACS, this research shows that in addition to 13% of persons with disability, there is another 13% of the population without disability in households with disability. Data from the ACS therefore suggest that over one-fourth of the U.S. population is impacted by disability, either of their own or a household member. However, disability estimates from the ACS, a general population survey, are deemed to be relatively conservative compared to surveys specific to health and disability (39) such as the Behavioral Risk Factor Surveillance System BFRSS of the Center for Disease Control CDC (30) or the National Health Interview Survey NHIS (40). The combined share of PwD and PwoD_HHwD is most likely much higher than one quarter. In addition, as the ACS is a cross-sectional survey giving a snapshot in time, an even higher share of the U.S. population may experience household-level disability over a lifetime. In contrast to the widespread impacts of disability in a household, disability does not receive all that much attention in public discourse. Even though disability is experienced by a sizeable share of the population, the perception that disability only involves a relatively small minority stems perhaps from limited visibility of persons with disability and their household members. Barriers limiting activities and constraining community participation could be contributing to this low visibility.

This research capitalizes on ACS microdata showing relationships within households and therefore information on household compositions. Importantly, it reveals the greater complexity of households with disability than those without disability, with the reference persons' sons/daughters without disability playing an important role in these households. The multigenerational makeup stands out as a feature of family households with disability. While there is a stark difference in disability rates between the most urban and most rural areas, ranging from 11 to 18%, urban–rural differences in the composition of households with disability are more modest. There are, however, some noteworthy differences for larger metro areas, with higher shares of multigeneration households. Greater ethnic diversity and the benefits of resource sharing in high-cost large cities may contribute to the larger share of multigenerational households.

Theories providing a background for this work address household decision-making (3–6), solidarity (10–12), and the capability-functioning nexus (15, 18–20, 22, 41). Solidarity-driven decisions of persons without disability contribute to a boost in household resources or commodities and favorably affect the practical opportunities or capabilities of persons with disability. A broader set of choices makes for increased functioning and reduced limitations on activities leading to broader opportunities to participate in the community outside the home. Persons with disability likely benefit from the presence of household members without disability who act to facilitate greater community participation. Concurrently, the desire for and benefits of community participation may be diminished as some of the social, emotional, and other needs may be met in the household due to the presence and support of other household members.

This raises the question, of course, of how community participation is or is not facilitated for the large number of solo persons with disability. Solo persons with disability may have a greater need but fewer opportunities for community participation. Are their needs met and if so how? This is especially relevant in rural areas where a third of persons in solo households experience disability.

Numeric results from the ACS provide a detailed picture of residential settings, disability, and household composition. Findings align with the literature on household decision-making, solidarity, and capabilities. However, the ACS is not designed to and does not provide explicit motivational and behavioral information. Qualitative research would be needed to specifically explore the nature of decision-making and support relationships and the linkages to capabilities, functioning, and community participation of persons with disability and members of their households. A qualitative research approach could also reveal how this plays out similarly or differently for persons with disability and their household members without disability. Does the presence of PwoD_HHwD raise the collective capabilities of the entire household, or is this more of a zero-sum game based on trade-offs between PwoD_HHwD and PwD? Such insight could have important implications, especially for policy recognizing and addressing the role of persons without disability in households with disability.

## Data availability statement

Publicly available datasets were analyzed in this study. This data can be found here: https://www2.census.gov/programs-surveys/acs/data/pums/2019/5-Year/.

## Author contributions

The author conceptualized and designed the study, conducted the data analysis, and wrote the manuscript.

## Funding

The content of this research was developed under grants from the National Institute on Disability, Independent Living, and Rehabilitation Research (NIDILRR Grant Numbers #90RT5023 and 90RTCP0002). NIDILRR is a Center within the Administration for Community Living (ACL), Department of Health and Human Services (HHS).

## Conflict of interest

The author declares that the research was conducted in the absence of any commercial or financial relationships that could be construed as a potential conflict of interest.

## Publisher's note

All claims expressed in this article are solely those of the authors and do not necessarily represent those of their affiliated organizations, or those of the publisher, the editors and the reviewers. Any product that may be evaluated in this article, or claim that may be made by its manufacturer, is not guaranteed or endorsed by the publisher.

## Author disclaimer

The findings and conclusions are those of the author and do not necessarily reflect the view or policy of NIDILRR, ACL, or HHS. Endorsement by the U.S. Federal Government should not be assumed.

## Abbreviations

RRID:SCR_011587 (U.S. Census Bureau): Resource Identification Initiative.
